# Comparison between scaling-root-planing (SRP) and SRP/photodynamic therapy: six-month study

**DOI:** 10.1186/1746-160X-8-12

**Published:** 2012-04-05

**Authors:** Mohammad Berakdar, Angelika Callaway, Mohammad Fakhr Eddin, Armin Roß, Brita Willershausen

**Affiliations:** 1 Department of Operative Dentistry, University Medical Center of the Johannes Gutenberg University Mainz, Germany

**Keywords:** Chronic periodontitis, Split mouth design, Photodynamic therapy, Scaling and root planing

## Abstract

**Introduction:**

The purpose of this long-term clinical study was to examine the additional efficacy of photodynamic therapy (PDT) to scaling and root planing (SRP) in patients with chronic periodontal disease.

**Methods:**

A total of 22 patients (mean age: 59.3 ± 11.7 years) with chronic periodontal disease and four teeth with probing depth ≥ 5 mm were enrolled in the study. Inclusion criteria were: no systemic disease, no smoking, no pregnancy and no long-term medication. Beside the anamnesis, the following clinical parameters were assessed at baseline (one week before therapy), and one, three and six months after the therapy: bleeding on probing (BOP), plaque index (PI) probing depth (PD), and clinical attachment loss. All measurements were done by the same examiner with a fixed periodontal probe (PCP 12, Hu-Friedy) at six measurements/tooth. In each patient, two teeth were treated with SRP alone and two teeth with SRP and PDT (Periowave, Ondine Biopharma, Vancouver, Canada). The nonparametric Wilcoxon test for paired samples was used for comparison of the effect of the two treatments (p ≤ 0.05).

**Results:**

After both types of treatment, the number of teeth positive for BOP declined. At baseline, the CAL measured 7.2 ± 1.2 mm (SRP) or 8.1 ± 1.3 mm (SRP/PDT); one, three and six months after both types of treatment an improvement was observed. At baseline, the probing depth was 5.9 ± 0.8 mm (SRP) or 6.4 ± 0.8 mm (SRP/PDT); after six months, an improvement of 2.4 ± 0.6 mm (SRP) or 2.9 ± 0.8 mm (SRP/PDT) was found. The greater reduction of the PD, achieved by a combination of SRP/PDT, was statistically significant after six months (p = 0.007).

**Conclusion:**

This clinical study demonstrates that SRP in combination with PDT seems to be effective and is therefore suitable as an adjuvant therapy to the mechanical conditioning of the periodontal pockets in patients with chronic periodontal diseases.

## Background

With respect to the etiology of inflammatory periodontal diseases, fundamental importance is attached to microbial plaque [1,2]. The pathogenic potential of the bacteria within the subgingival plaque influences the progression and severity of the periodontal disease, which - in most cases - is accompanied by destruction of periodontal ligament and alveolar bone [3]. However, in addition to the causal microbial factors, especially in chronic periodontitis other risk factors e.g. smoking, cardiovascular disease, diabetes mellitus as well as other systemic conditions or hematologic or immunologic disorders can have a crucial significance [4]. The primary goal of the therapy of inflammatory periodontal diseases to stop the progression of the disease is the reduction or destruction of the microbial biofilm, including the micro-organisms attached to the root surfaces. Here the biofilm also has a key function, because it provides a variety of bacteria with especially good symbiotic growth conditions. An effective reduction of the subgingival biofilm can be achieved by employing various techniques [5,6]. However, important for a lasting success of a treatment is a continuous supportive periodontal therapy employing individual prophylactic measures and regular professional tooth cleaning [7].

In addition to the classical method of using hand instruments for reduction of the bacterial load and root planing, various other techniques have been established including the usage of ultrasonic devices or lasers. Many attempts have been made over time to increase the efficacy and efficiency of subgingival debridement and bacterial elimination [8,9]. Hand instrumentation is still considered the gold standard and allows the sufficient cleaning of the periodontal pockets [10]. Anatomical peculiarities like root curvatures or invaginations can make it difficult to remove bacterial deposits and biofilms completely from root surfaces by means of mechanical methods [11,12]. Several treatment options are available to support the efficacy of instrumentation, for example the usage of local antibiotics or antimicrobials [13] or photodynamic therapy (PDT).

Photodynamic therapy combines low level laser light with a photosensitizer (a non-toxic dye), which binds to the target cells. Photosensitizers, like for example toluidine blue O, methylene blue and malachite green, absorb light of a specific wavelength. In the excited state theses molecules can react with molecules from the environment, for example with oxygen. Reactive oxygen species can be generated, which can cause oxidative damage to the target cells [14]. Several photosensitizers are available which work in combination with a low level laser (wave length of λ = 630-670 nm) to destroy microorganisms [15]. Therefore, photodynamic therapy can be an alternative for reducing the bacterial load in periodontal pockets [16]. In vitro studies have shown the effectiveness of photodynamic therapy against bacteria from subgingival plaque samples from patients with chronic destructive periodontitis [15] and also against *Aggregatibacter actinomycetemcomitans *[17]. In an animal model, the effectiveness of PDT in suppressing *Porphyromonas gingivalis *and reducing gingival inflammation was demonstrated [18]. A recent clinical study reported a beneficial effect of PDT, alone or in combination with SRP, on pocket probing depth reduction and improvements of clinical attachment level and bleeding on probing scores [19]. However, another study found statistically significant differences only for reduction of full-mouth bleeding scores, when comparing treatments with and without PDT as adjunct, but not for improvements of probing depth and clinical attachment level [20]. There are still only limited data available from controlled clinical studies evaluating the effects of PDT as an adjunct to SRP in supportive periodontal treatment [21].

The aim of the present study was therefore to examine in patients with chronic periodontitis an efficacy of the photodynamic therapy in addition to the classical treatment with scaling und root planing. Treatments were performed using the split mouth design, and the patients were examined at recall visits covering a period of six months.

## Materials and methods

### Patients

A total of 22 adults, aged 38 to 74 years, presented as outpatients to the Department of Operative Dentistry of the University Medical Center, Johannes Gutenberg University Mainz, and were enrolled in the present study. All patients (10 = female; 12 = male) were diagnosed with chronic periodontitis, with four teeth having at least one site with a probing depth of five mm, and presence of bleeding on probing (BOP). The subjects were informed in detail about the aim and course of the study and gave written informed consent. The approval of the course of the study by the ethics commission Mainz (number: 837.132.08 (6129)) was obtained before the beginning of this study. Criteria for exclusion from this study were: presence of a systemic disease, treatment with antibiotics within the last six months, pregnancy, and smoking. For inclusion in the study, the patients had to have at least four teeth with a probing depth of ≥ 5 mm. In addition, a good patients' compliance was required, which was monitored over the course of the study by means of measuring plaque and gingival indices. At the beginning of the study, two types of therapy were selected: scaling and root planing (SRP) or SRP and photodynamic therapy (SRP + PDT). For each patient it was decided by means of a randomization list, which tooth was to receive which type of therapy. The treatment was done according to a "split mouth design", so that in each patient two teeth belonged to the control group (SRP) and two to the test group (SRP + PDT). All subsequent examinations were done by the same examiner with a fixed periodontal probe (PCP 12, Hu-Friedy, Chicago, IL, USA). All patients received a professional tooth cleaning three weeks prior to the treatment begin. The measurements of the clinical parameters were performed at baseline (one week before treatment), and one month, three and six months after treatment.

### Clinical parameters

At each visit, probing depths, absence or presence of bleeding on probing (BOP), gingival recessions and clinical attachment levels at six sites per tooth (buccal; mesiobuccal; distobuccal; lingual; mesiolingual; distolingual) were recorded by the examiner. The examiner was not involved in the therapy, and therefore didn't know which tooth had received which type of therapy (single blinded). To assess the patients' compliance, the gingival index [GI, Löe and Silness] and the plaque index [PI, Silness and Löe] were determined in addition.

### Course of treatment

Scaling and root planing was performed in all 22 patients by the same examiner with hand instruments (Gracey curettes, Hu-Friedy, Chicago, IL, USA). Teeth belonging to the test group received a photodynamic therapy in addition. For the photodynamic therapy 0.005% methylene blue was used as photosensitizer and activated with a laser [Periowave, Ondine Biopharma, Vancouver, Canada] at a wavelength of 670 nm and a maximum power of 150 mW for 60 seconds. All patients were assessed again by the same examiner at recall visits one month, three and six months after treatment.

### Statistical analysis

The statistical analysis of the data was performed in collaboration with the Institute of Medical Biostatistics, Epidemiology and Informatics of the University Medical Center of the Johannes Gutenberg University Mainz, using the program SPSS for medical statistics (17.0 for Windows, Chicago, IL, USA). Descriptive statistics were calculated, and values are given as means ± SD or are shown as boxplots. A descriptive analysis of the gain in clinical attachment and the improvement in probing depths was performed. Comparisons were made for the two different treatments (SRP or SRP + PDT), using as nonparametric test the Wilcoxon test for paired samples. The significance level was set at p ≤ 0.05.

## Results

**Figure 1:**
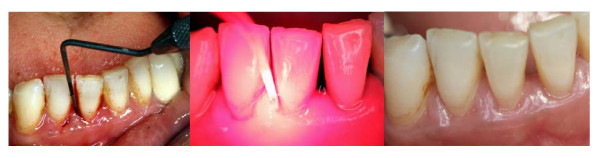
**Patient with chronic periodontitis before treatment (left), during photodynamic therapy (middle) and six months after treatment (right)**.

All subjects, enrolled in the present study as outpatients, with a mean age of 59.3 years (SD: 11.7 years), could be examined as planned one month, three and six months after the end of the periodontal treatment. In each case, the chronic periodontitis could be treated successfully by means of the two different therapy concepts using a split mouth design, as it had been explained to the patients (Figure 1). No undesirable effects were observed, and both therapies were tolerated well by the patients. Both therapies lead to a significant reduction in the number of teeth positive when tested for bleeding on probing (BOP), as is shown in Table 1.

**Table 1:** Frequency (%) of cases (n = 22, split mouth design) with at least one tooth positive for bleeding on probing (BOP)

Therapy	SRP	SRP + PDT
	
	BOP positive	BOP positive
at baseline	100%	100%

after one month	27.3%	68.2%

after three months	27.3%	22.7%

after six months	22.7%	13.6%

**Figure 2:**
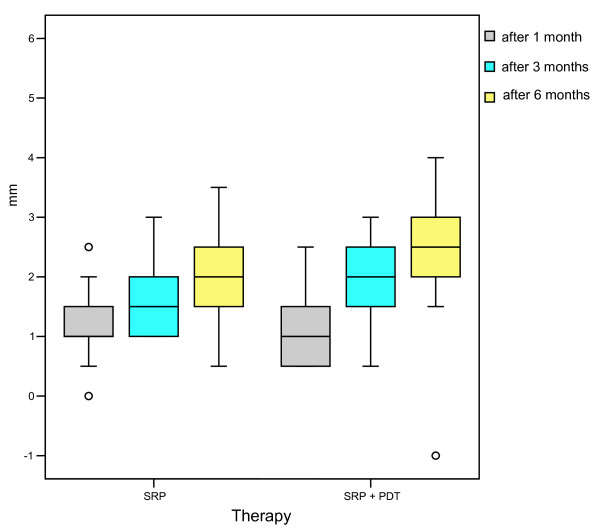
**Gain in the clinical attachment (mm) one month, two and three months after therapy with scaling and root planing alone (SRP) or in combination with photodynamic therapy (SRP + PDT), n = 22**.

The scores for the plaque index (PI, scores 0-3) were similar for both therapy forms, both before treatment and at the end of the study. At baseline, all teeth showed high scores for the plaque index; 73% of the patients had a score of 3. One month after treatment, considerably lower plaque index scores were determined for both therapy forms. After three months, 77% of the teeth treated with SRP alone had low scores of 0 or 1 for the plaque index, and after the combined therapies of SRP and PDT, in 82% of the teeth scores of 0 or 1 were found for the plaque index. After six months, a slight increase in the plaque index scores was observed; however, independent of the type of treatment, in none of the cases a high plaque index score of 3 was determined. Similar results were found with regard to the measurements of the clinical attachment levels (CAL) over a period of six months. At baseline, the CAL measured 7.2 ± 1.2 mm (SRP) or 8.1 ± 1.3 mm (SRP + PDT). Both therapies lead to a recognizable improvement of the CAL values, with the combined therapy achieving a slightly higher gain in clinical attachment (Figure 2). At the end of the observation period of six months, there was a clear difference in the effect of the two therapies (p = 0.052).

**Figure 3:**
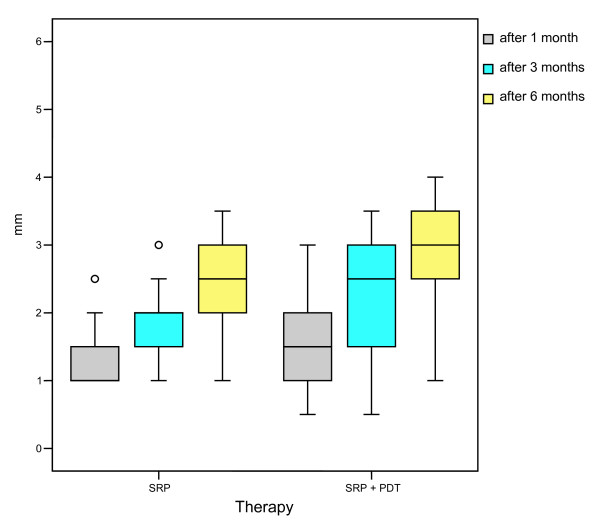
**Improvement of probing depths (mm) one month, two and three months after therapy with scaling and root planing alone (SRP) or in combination with photodynamic therapy (SRP + PDT), n = 22**.

At baseline, the probing depth was 5.9 ± 0.8 mm (SRP) or 6.4 ± 0.8 mm (SRP/PDT). Both after the treatment with SRP alone, and with the combined treatment with SRP, followed by photodynamic therapy (PDT), a clear improvement in the measured probing depths was observed over an observation period of six months. As is shown in Figure 3, already after four weeks for SRP therapy alone a mean reduction of the pocket depths by 1.3 ± 0.4 mm, and for the combination therapy of SRP + PDT a mean decrease in the probing depths of 1.5 ± 0.6 mm was found. However, after an observation period of six months, the combined therapy SRP + PDT showed with a mean reduction in probing depths of 2.9 ± 0.8 mm a statistically significant improvement (p = 0.007), in comparison to SRP therapy alone (2.4 ± 0.6 mm).

## Discussion

Treatment concepts of non-surgical therapy for chronic periodontitis comprise as an essential parameter the reduction of periodontal pathogens. In addition to the classical treatment using scaling and root planing (SRP), nowadays adjuvant low-level laser therapies together with dyes as photosensitizer are available, which are however partly being discussed controversially. Most studies examine the effectiveness of the adjuvant photodynamic therapy (PDT) only for a short period of time; therefore, in the present clinical study the possible additional success of the photodynamic therapy as adjunct to SRP in comparison to treatment with SRP alone was examined over a period of six months.

The split mouth design was chosen for the present study, and all patients were examined at baseline, and then the various clinical parameters were re-evaluated one, three and six months after treatment.

The plaque index improved equally at all sites from baseline to the final assessment six months after treatment, which supports the decision for using a split mouth design for the present study. In addition, the repeated evaluation of the plaque scores can be seen as a tool to monitor patient compliance. Miyamoto et al. [22] examined the role of patient compliance for the success of periodontal therapy over a long-tem observation period of 15 to 23 years. They measured clinical parameters including probing depth, bleeding on probing, plaque index and tooth loss. Patient compliance was defined as the frequency of dental visits, and it could be shown that the patients who attended most of the scheduled dental visits also had the highest reduction in plaque scores in comparison to the other patients.

Lang et al. [23] emphasized the importance of the bleeding on probing index as predictor for the progression of periodontal disease. The decrease in BOP incidence in the present study reflects the observation by Lang et al. [23] that a reduction in BOP scores is accompanied by a decrease in periodontal inflammation.

In the present study, the clinical attachment level improved significantly, both in the sites treated with SRP alone and in those treated with a combination of SRP and PDT, from baseline to the final examination. The differences in outcome between the two treatments tended to be greater for the sites treated with SRP and PDT. Similar improvements were also seen concerning the reduction in probing depth, which was after six months statistically significantly higher for the sites treated with SRP and PDT. Andersen et al. [19], who examined the influence of SRP alone or in combination with PDT on clinical parameters, found similar results. After an observation period of 12 weeks, higher improvement of the clinical parameters probing depth and CAL was found for the additional PDT therapy.

Chondros et al. [21] examined 24 patients over a period of six months, and examined the clinical and microbiological effect of PDT as adjunct to SRP versus treatment with SRP alone. Improvements in probing depth, CAL and of FMPS (full mouth plaque score) were found; however, there were no statistically significant differences between the two groups. In contrast, a statistically significant higher decrease in BOP scores was found for the group treated with the combined therapies. The review by Woodruff et al. [24] supports the beneficial effect of low-level laser therapy on tissue healing, including augmentation of collagen synthesis, reduced healing time and diminution of the size of the wound.

Yilmaz et al. [25], however, found little influence of an additional treatment with PDT to SRP. They showed that in comparison to the sole SRP therapy there were no significant differences in the improvement of the clinical and microbiological parameters, evaluated over an observation period of 32 days. Similar results are reported by Polansky et al. [26], who didn't find any additional effect on the reduction of probing depth and BOP scores, when subgingival ultrasonic treatment was combined with PDT.

A slight effect of an adjunct PDT to SRP could be demonstrated by Ge et al. [5]. They could show that over a period of three months the group of patients treated with the combined therapy showed lower BOP scores than the group treated with SRP alone, while the improvement of other clinical parameters like probing depth and CAL didn't differ between groups.

De Oliveira et al. [27] studied the effect of treatment with PDT or SRP on the cytokine profiles of 10 patients with aggressive periodontitis. After a period of 90 days, a significant reduction in the cytokine levels was found, but there was no difference between the two types of treatment. In contrast, Liu et al. [28] could show over an observation period of four weeks that after an additional treatment with PDT to SRP therapy a significantly greater reduction of the interleukin-1β concentration in gingival crevicular fluid occurred. In this study with 24 patients using a split mouth design, also a higher reduction of the probing depth and also in the BOP scores was found after four weeks for the sites treated with the combined therapy. Rühling et al. [29] found in residual pockets of 54 patients, treated either with PDT or with an ultrasonic scaler, over a period of three months a reduction of probing depth, but the difference between the two groups was not statistically significant. Braun et al. [30] studied the clinical effects of PDT as supportive therapy to SRP in 20 patients. The patients were treated using a split mouth design and were assessed over a period of three months. Both therapies led three months after treatment to significant reductions in probing depth, sites positive for BOP and relative attachment level in comparison to the values at baseline. As in the present study, a higher reduction in probing depth was achieved for sites treated with the combined therapy, and the difference to treatment with SRP only was also statistically significant.

In contrast, in a study on 33 patients with chronic periodontitis, over an observation period of six months no additional effect of an adjunct PDT versus SRP alone was found with respect to clinical parameters including probing depth, BOP or CAL [31]. However, a statistically significantly greater reduction for sites positive for various periodontal pathogens, including *Aggregatibacter actinomycetemcomitans, Porphyromonas gingivalis, Prevotella intermedia, Prevotella nigrescens *and *Tannerella forsythia*, was found after adjunct PDT in comparison to SRP alone.

In the presence of anatomical structures like dental furcations or invaginations, access to the sites for a complete removal of subgingival biofilm with hand instruments might be difficult. In such cases, the antimicrobial effect of PDT might help achieve a better healing of periodontal defects. De Almeida et al. [32] used Wistar rats in an animal model to induce experimental periodontitis at the first mandibular molar. The animals were treated with methylene blue, laser or PDT, and bone loss in the furcation area was examined by histometric analysis. It was shown that the amount of bone loss was lowest in the group treated with PDT, and the difference to all other groups was statistically significant.

## Conclusions

In conclusion it can be confirmed that the photodynamic therapy as adjunct to classical scaling and root planing can be recommended as treatment option, which can by no means replace the classical therapy concepts. But even over an observation period of six months a slightly higher improvement of the clinical parameters was achieved than with SRP alone.

## Competing interests

The authors declare that they have no competing interests.

## Authors' contributions

MB and MFE carried out the study. AC and MFE performed the statistical analysis. MB, AC, MFE, AR and BW conceived of the study, and participated in its design and coordination. All authors read and approved the final manuscript.

## References

[B1] Löe H Theilade E Jensen SB Experimental gingivitis in man J Periodontol 1965 36 177 187 10.1902/jop.1965.36.3.177 14296927

[B2] Theilade E Wright WH Jensen SB Löe H Experimental gingivitis in man. II. A longitudinal clinical and bacteriological investigation J Periodontol Res 1966 1 1 13 10.1111/j.1600-0765.1966.tb01842.x 4224181

[B3] Page RC Offenbacher S Schroeder H Seymour GJ Kornman KS Advances in the pathogenesis of periodontitis: summary of developments, clinical implications and future directions Periodontol 2000 1997 4 216 248 9567973 10.1111/j.1600-0757.1997.tb00199.x

[B4] Nunn ME Understanding the etiology of periodontitis: an overview of periodontal risk factors Periodontol 2000 2003 32 11 23 10.1046/j.0906-6713.2002.03202.x 12756030

[B5] Ge L Shu R Li Y Li C Luo L Song Z Xie Y Liu D Adjunctive effect of photodynamic therapy to scaling and root planing in the treatment of chronic periodontitis Photomed Laser Surg 2011 29 33 37 10.1089/pho.2009.2727 21166588

[B6] Oda S Nitta H Setoguchi T Izumi Y Ishikawa I Current concepts and advances in manual and power-driven instrumentation Periodontology 2000 2004 36 45 58 10.1111/j.1600-0757.2004.03674.x 15330943

[B7] Axelsson P Lindhe J Effect of controlled oral hygiene procedures on caries and periodontal disease in adults. Results after 6 years J Clin Periodontol 1981 8 239 248 10.1111/j.1600-051X.1981.tb02035.x 6947990

[B8] Schwarz F Sculean A Berakdar M Georg T Reich E Becker J Periodontal treatment with an Er:YAG laser or scaling and root planing. A 2-year follow-up split-mouth study J Periodontol 2003 74 590 596 10.1902/jop.2003.74.5.590 12816290

[B9] Tunkel J Heinecke A Flemmig TF A systematic review of efficacy of machine-driven and manual subgingival debridement in the treatment of chronic periodontitis J Clin Periodontol 2002 29 72 81 10.1034/j.1600-051X.29.s3.4.x 12787208

[B10] Cugini MA Haffajee AD Smith C Kent RL Socransky SS The effect of scaling and root planing on the clinical and microbiological parameters of periodontal diseases: 12-month results J Clin Periodontol 2000 27 30 36 10.1034/j.1600-051x.2000.027001030.x 10674959

[B11] Petersilka GJ Draenert M Mehl A Hickel R Flemming TF Safety and efficiency of novel sonic scaler tips in vitro J Clin Periodontol 2003 30 551 555 10.1034/j.1600-051X.2003.00300.x 12795794

[B12] Tomasi C Schander K Dahlen G Wennström JL Short-term clinical and microbiologic effects of pocket debridement with an Er:YAG laser during periodontal maintenance J Periodontol 2006 77 111 118 10.1902/jop.2006.77.1.111 16579711

[B13] Etienne D Locally delivered antimicrobials for the treatment of chronic periodontitis Oral Dis 2003 9 45 50 10.1034/j.1601-0825.9.s1.8.x 12974530

[B14] Sharman WM Allen CM van Lier JE Photodynamic therapeutics: basic principles and clinical applications Drug Discov Today 1999 4 507 517 10.1016/S1359-6446(99)01412-9 10529768

[B15] Soukos NS Mulholland SE Socransky SS Doukas AG Photodestruction of human dental plaque bacteria: enhancement of the photodynamic effect by photomechanical waves in an oral biofilm model Las Surg Med 2003 33 161 168 10.1002/lsm.10208 12949945

[B16] Wilson M Dobson J Harvey W Sensitization of oral bacteria to killing by low-power laser radiation Curr Microbiol 1992 25 77 81 10.1007/BF01570963 1369193

[B17] Prates RA Yamada AM Jr Suzuki LC Eiko Hashimoto MC Cai S Gouw-Soares S Gomes L Ribeiro MS Bactericidal effect of malachite green and red laser on Actinobacillus actinomycetemcomitans J Photochem Photobiol B 2007 86 70 76 10.1016/j.jphotobiol.2006.07.010 16979345

[B18] Sigusch BW Pfitzner A Albrecht V Glockmann E Efficacy of photodynamic therapy on inflammatory signs and two selected periodontopathogenic species in a beagle dog model J Periodontol 2005 76 1100 1105 10.1902/jop.2005.76.7.1100 16018752

[B19] Andersen R Loebel N Hammond D Wilson M Treatment of periodontal disease by photodisinfection compared to scaling and root planing J Clin Dent 2007 8 34 38 17508621

[B20] Christodoulides N Nikolidakis D Chondros P Becker J Schwarz F Rössler R Sculean A Photodynamic therapy as an adjunct to non-surgical periodontal treatment: a randomized, controlled clinical trial J Periodontol 2008 79 1638 1644 10.1902/jop.2008.070652 18771363

[B21] Chondros P Nikolidakis D Christodoulides N Rössler R Gutknecht N Sculean A Photodynamic therapy as adjunct to non-surgical periodontal treatment in patients on periodontal maintenance: a randomized controlled clinical trial Lasers Med Sci 2009 24 681 688 10.1007/s10103-008-0565-z 18465191

[B22] Miyamoto T Kumagai T Jones JA Van Dyke TE Nunn ME Compliance as a prognostic indicator: retrospective study of 505 patients treated and maintained for 15 years J Periodontol 2006 77 223 232 10.1902/jop.2006.040349 16460248

[B23] Lang NP Joss A Orsanic T Gusberti FA Siegrist BE Bleeding on probing. A predictor for the progression of periodontal disease? J Clin Periodontol 1986 13 590 596 10.1111/j.1600-051X.1986.tb00852.x 3489010

[B24] Woodruff LD Bounkeo JM Brannon WM Dawes KS Barham CD Waddell DL Enwemeka CS The efficacy of laser therapy in wound repair: a meta-analysis of the literature Photomed Laser Surg 2004 22 241 247 10.1089/1549541041438623 15315732

[B25] Yilmaz S Kuru B Kuru L Noyan Ü Argun D Kadir T Effect of gallium arsenide diode laser on human periodontal disease: a microbiological and clinical study Lasers Surg Med 2002 30 60 66 10.1002/lsm.10010 11857606

[B26] Polansky R Haas M Heschl A Wimmer G Clinical effectiveness of photodynamic therapy in the treatment of periodontitis J Clin Periodontol 2009 36 575 580 10.1111/j.1600-051X.2009.01412.x 19554711

[B27] de Oliveira RR Schwartz-Filho HO Novaes AB Garlet GP de Souza RF Taba M Scombatti de Souza SL Ribeiro FJ Antimicrobial photodynamic therapy in the non-surgical treatment of aggressive periodontitis: cytokine profile in gingival crevicular fluid, preliminary results J Periodontol 2009 80 98 105 10.1902/jop.2009.070465 19228095

[B28] Lui J Corbet EF Jin L Combined photodynamic and low-level laser therapies as an adjunct to nonsurgical treatment of chronic periodontitis J Periodont Res 2011 46 89 96 10.1111/j.1600-0765.2010.01316.x 20860592

[B29] Rühling A Fanghänel J Houshmand M Kuhr A Meisel P Schwahn C Kocher T Photodynamic therapy of persistent pockets in maintenance patients-a clinical study Clin Oral Invest 2010 14 637 644 10.1007/s00784-009-0347-4 19823880

[B30] Braun A Dehn C Krause F Jepsen S Short-term clinical effects of adjunctive antimicrobial photodynamic therapy in periodontal treatment: a randomized clinical trial J Clin Periodontol 2008 35 877 884 10.1111/j.1600-051X.2008.01303.x 18713259

[B31] Theodoro LH Silva SP Pires JR Soares GH Pontes AE Zuza EP Spolidorio DM de Toledo BE Garcia VG Clinical and microbial effects of photodynamic therapy associated with nonsurgical periodontal treatment. A 6-month follow-up Lasers Med Sci 2011 in press 10.1007/s10103-011-0942-x 21687979

[B32] de Almeida JM Theodoro LH Bosco AF Nagata MJH Oshiiwa M Garcia VG In vivo effect of photodynamic therapy on periodontal bone loss in dental furcations J Periodontol 2008 79 1081 1088 10.1902/jop.2008.070456 18533787

